# Which obesity phenotypes predict poor health-related quality of life in adult men and women? Tehran Lipid and Glucose Study

**DOI:** 10.1371/journal.pone.0203028

**Published:** 2018-09-12

**Authors:** Parisa Amiri, Sara Jalali-Farahani, Marjan Rezaei, Leila Cheraghi, Farhad Hosseinpanah, Fereidoun Azizi

**Affiliations:** 1 Research Center for Social Determinants of Health, Research Institute for Endocrine Sciences, Shahid Beheshti University of Medical Sciences, Tehran, Iran; 2 Students’ Research Committee, Shahid Beheshti University of Medical Sciences, Tehran, Iran; 3 Department of Biostatistics and Epidemiology, Research Institute for Endocrine Sciences, Shahid Beheshti University of Medical Sciences, Tehran, Iran; 4 Obesity Research Center, Research Institute for Endocrine Sciences, Shahid Beheshti University of Medical Sciences, Tehran, Iran; 5 Endocrine Research Center, Research Institute for Endocrine Sciences, Shahid Beheshti University of Medical Sciences, Tehran, Iran; University of Adelaide School of Medicine, AUSTRALIA

## Abstract

**Purpose:**

This study aimed to explore the association between different obesity phenotypes and health-related quality of life (HRQoL) among Tehranian men and women.

**Methods:**

The participants of this study were 2880 healthy adults (aged>19 years) who participated in Tehran Lipid and Glucose Study (TLGS). To obtain socio-demographic and HRQoL information, participants were interviewed by trained interviewers and were stratified by body mass index categories and metabolic status. Dysmetabolic status was defined as having either metabolic syndrome or diabetes according to the Joint Interim Statement definition and American Diabetes Association. Poor HRQoL was defined as the first quartile of HRQoL scores and logistic regression analysis was used to compare sex-specific odds ratios.

**Results:**

Mean age of participants was 47.7±15.6 and 47.8±14.2 years in men and women respectively. The most and the least common obesity phenotypes were overweight-normal metabolic status and normal weight-dysmetabolic status, respectively. Only mean scores for physical HRQoL were significantly different among obesity phenotypes in both men and women (p<0.05). In addition, after adjusting for age, marital status, level of education, job status and physical activity, the odds of reporting poor physical HRQoL was significantly higher in men (OR: 1.960, 95% CI: 1.037–3.704; p<0.05) and women (OR: 2.887, 95% CI: 1.674–4.977; p<0.001) with obese-dysmetabolic status, compared to their counterparts with normal weight-normal metabolic status. However, except for overweight-normal metabolic women, who were less likely to report poor mental HRQoL (OR: 0.638, 95% CI: 0.415–0.981; p<0.05), none of the phenotypes were associated with poor mental HRQoL in either gender.

**Conclusions:**

Compared to those with normal weight normal metabolic status, only obese dysmetabolic individuals were more likely to report poor physical HRQoL in both genders.

## Introduction

Obesity has become a major public health issue over the past few decades and its prevalence is on the rise worldwide [1]. Along with the increasing worldwide trend of obesity, a considerable increase in overweight and obesity has also been observed in Iran [2,3]. A recent systematic review conducted on Iranian adults reports average prevalence ranges of 27.0–38.5% and 12.6–25.9% for overweight and obesity respectively [3]. Although a large body of evidence shows that obesity could be a trigger point for many chronic diseases such as diabetes, cancer, cardiovascular diseases (CVD) and all-cause mortality [4–7], there is much data indicating that beyond body mass index (BMI) status, combining obesity with metabolic health components yields a spectrum of obesity phenotypes which can lead to different cardiovascular outcomes [8–11].

There are controversial data regarding the predisposing role of different obesity phenotypes, specifically among those recognized as metabolically healthy obese or metabolically abnormal normal weight individuals in developing CVD and other related objective outcomes [11–16]. Despite a number of studies reporting metabolically healthy obesity as a relatively safe condition in regard to developing CVD [12,13,17,18], results of other studies cast doubt on the concept of benign metabolically healthy obese and suggest that in the long term the risk of developing CVD in metabolically healthy obese phenotype is on the rise [11,14,19]. In spite of much data comparing the prognostic impact of different obesity phenotypes on measurable objective outcomes, there is currently limited data comparing the diagnostic significance of these phenotypes in detecting poor health-related quality of life (HRQoL) as a subjective patient-centered outcome.

The association between obesity and HRQoL, defined as the individual’s subjective evaluation of their physical and mental wellbeing is well documented. While most studies report the adverse associations between overweight and HRQoL mainly in the physical domain [20–22] there are limited findings available indicating that both the physical and mental aspects of HRQoL are adversely affected by excessive weight gain in different populations [21,23]. In this regard a meta-analysis showed that physical HRQoL could be reduced in adults with different categories of obesity, although mental HRQoL was only affected by severe obesity status [24]. Another recent study from Iran also showed the adverse relation of overweight and obesity with HRQoL, mainly in physical domain [25].

Despite previous evidence confirming the relation of obesity [20–28] and cardio-metabolic risk factors [29–33] with different aspects of HRQoL, only few studies have investigated the cumulative influence of weight and metabolic conditions on the self-evaluated health status of participants. In this regard the association between metabolic syndrome (a cluster of cardio metabolic risk factors including central obesity) and HRQoL, has been previously demonstrated [34–36]; however, there are only three studies that have focused on the relation of HRQoL with obesity phenotypes, with different metabolic conditions and levels of general obesity [37–39]; in this regard, one cross sectional study, conducted on a Scottish population showed an independent metabolic association between HRQoL and obesity [37]. Contrary to this finding, two other studies conducted on Spanish and Korean adults suggest that, compared to metabolically abnormal-normal weight individuals, both metabolically healthy-obese and metabolically unhealthy-obese phenotypes are more likely to report poor physical HRQoL [38,39]. Although the association between HRQoL and obesity phenotypes has been investigated previously in the above mentioned studies, little is known about subjective health evaluation among different obesity phenotypes in Middle-Eastern populations. Considering the fact that subjective perceptions of health and wellbeing could be influenced by individuals’ characteristics and also their socio-cultural environment [40], investigating the relation between HRQoL and obesity phenotypes in these countries could provide a more informative picture in this regard. In this study we aimed to evaluate the impact of HRQoL on different obesity phenotypes in Tehranian adults, participating in the Tehran Lipid and Glucose Study (TLGS).

## Materials and methods

### Participants

The participants of this study were adults (aged >19 years), recruited from the 6^th^ phase of the TLGS (2014–2016), an ongoing community-based study being conducted to determine the risk factors and prevent non-communicable diseases among residents of district 13 of Tehran. District No. 13, one of the 22 districts of Tehran metropolitan city which is located in the Eastern part of the city covering an area of about 13 sq. kms, is under coverage of Shahid Beheshti University of Medical Sciences. At baseline a total of 15005 individuals, aged ≥3 years were selected using multistage cluster random sampling method and have been followed every 3 years. According to the baseline assessment, age distribution and socioeconomic status of the population in district No. 13 could be considered as representative of the overall population of Tehran at the beginning of the study (Iran National Census, 1996). Study details have been published previously [41].

For the current study, from among individuals who had participated in the TLGS during 2014–2016 (n = 3491), after excluding those with cancer, CVD and history of hospitalization (n = 415) and those with missing data on obesity phenotypes (n = 208), all adult participants (>19 years) who had complete data on SF-12 and obesity phenotypes were recruited (n = 2880). This study was approved by the research ethics committee of the Research Institute for Endocrine Sciences of the Shahid Beheshti University of Medical Sciences and all participants signed written informed consent forms, prior to data collection.

### Measurements

To obtain information about socio-demographic factors, smoking status, physical activity and HRQoL, participants were interviewed by trained interviewers, using validated questionnaires. Socio-demographic data and smoking habits were assessed, using a pretested questionnaire. Physical activity and HRQoL were assessed using the validated Iranian version of the Modifiable Activity Questionnaire (MAQ) [42] and the Short-Form 12-Item Health Survey version 2 (SF-12v2) respectively [43]. Levels of physical activity were calculated and reported as MET/Week and were categorized in three groups including: 1) Low physical activity: ≤ 600 MET/Week, 2) Moderate physical activity: >600 and ≤3000 MET/Week, 3) High physical activity: > 3000 MET/Week. Weight of participants was measured while they wore minimum clothing and without shoes, using a digital scale and recorded to the nearest 100 g. Height was measured in a standing position, without shoes and with shoulders in normal alignment. Waist circumference was measured using an unstretched tape meter and recorded to the nearest 0.1 cm. After a 15-minute rest, systolic and diastolic blood pressure were measured twice by a trained physician, using a standard mercury sphygmomanometer while participants were seated. After 12–14 hours of overnight fasting, blood samples were taken from all participants and the blood analyses were conducted on the day of blood collection. Further details on assessment of fasting blood sugar (FBS) and serum lipids (total cholesterol (TC), high density lipoprotein cholesterol (HDL-C) and triglycerides (TG)) have been provided previously [41].

### Definitions

Obesity phenotypes were determined by the weight and metabolic status of participants. To determine overweight and obesity, body mass index (BMI) were calculated, overweight and obesity were defined as having BMI between ≥25 and <30 kg/m^2^ and BMI of ≥ 30 kg/m^2^, respectively. Dysmetabolic status was defined as having either metabolic syndrome or diabetes according to the definition of the Joint Interim Statement (JIS) and the American Diabetes association (ADA) respectively. Based on the JIS definition, metabolic syndrome is defined as the presence of any three of the following five risk factors: 1) abdominal obesity, defined as waist circumference (WC) ≥90 cm for both genders in Iran [44,45]; 2) reduced HDL-C <50 mg/dl in women, <40 in men or on drug treatment for reduced HDL-C; 3) elevated triglycerides (TG) levels ≥ 150 mg/dl or on drug treatment for elevated TG; 4) elevated blood pressure (≥130 mmHg systolic blood pressure or ≥ 85 mmHg diastolic blood pressure) or on antihypertensive drug treatment for hypertension and 5) elevated fasting blood glucose (FBG)≥100 mg/dl or on drug treatment for elevated glucose levels [46]. Diabetes was defined as having fasting blood sugar ≥126 mg/dl or 2 hour post glucose ≥200 mg/dl or on medication for diagnosed diabetes. Six obesity phenotypes were defined, which included: 1) normal weight and normal metabolic status; 2) overweight and normal metabolic status; 3) obese and normal metabolic status; 4) normal weight and dysmetabolic status; 5) overweight and dysmetablic status; and 6) obese and dysmetabolic status.

### Statistical analysis

For continuous variables, means, standard deviation (SD) and for categorical ones, frequencies (percentage) are reported. For TG, median (Q_1_-Q_3_) are reported. Continuous and categorical variables were compared between men and women using t-test (Mann-Whitney test for TG) and Chi-square test respectively. To compare continuous and categorical variables among obesity phenotypes, ANOVA and chi-square test respectively were used. HRQoL mean scores were compared among obesity phenotypes using ANCOVA analysis and variables which significantly differed among obesity phenotypes were adjusted in the model.

Poor HRQoL was defined as the first quartile of physical component summary (PCS) and the mental component summary (MCS). To compute the odds ratios (ORs), logistic regression analysis was used. Sex specific ORs with 95% confidence intervals were computed and reported for men and women separately; model 1 was unadjusted, while model 2 was adjusted for age, marital status (Ref.: Married), education (Ref.: Higher), job status (Ref.: Employed) and physical activity (Ref: High). Statistical analysis was performed using SPSS software version 15 (SPSS Inc., Chicago, IL, USA), with significance set at p <0.05.

## Results

Mean age and BMI of participants were 47.8±14.8 years and 28.0±4.9 kg/m^2^ respectively. Descriptive statistics of participants are illustrated in Table 1. Most participants were married and had completed secondary education. In terms of job status, majority of men and women were employed and housewives respectively. Only 3.1% of women were smokers versus 19.3% of men. The prevalence of dysmetabolic status in men and women was 43.9% and 41.0% respectively. In men the prevalence of overweight and obesity were 47.1% and 23.7% and in women they were 38.6% and 35.1% respectively. The most and the least common obesity phenotypes were overweight-normal metabolic status (24.1% in men and 24.3% in women) and normal weight-dysmetabolic status (5.5% in men and 3.3% in women) respectively. The distribution of socio-demographic factors is shown in Table 2, indicating, significant differences in both genders in mean age and distribution of marital status, level of education, job status and physical activity among different obesity phenotypes.

**Table 1:** Descriptive statistics of study participants.

	Total (n = 2880)	Men (n = 1158)	Women (n = 1722)	**P value
**Age (years)**	47.8±14.8*	47.7±15.6	47.8±14.2	0.866
**Marital status n(%)**				
-Single	404(14.1)	210(18.2)	194(11.3)	<0.001
-Married	2244(78.1)	925(80.0)	1319(76.8)	
-Divorced/Widowed	225(7.8)	21(1.8)	204(11.9)	
**Level of education n(%)**				
-Primary	854(29.7)	282(24.4)	572(33.2)	<0.001
-Secondary	1111(38.7)	458(39.7)	653(38.0)	
-Higher	909(31.6)	414(35.9)	495(28.8)	
**Job status n(%)**				
- Unemployed/student/housewife	1334(46.4)	88(7.6)	1246(72.4)	<0.001
- Unemployed, but had other sources of income	410(14.3)	227(19.7)	183(10.6)	
- Employed	1130(39.3)	837(72.7)	293(17.0)	
**Smoking status**				
-Never	2346(81.7)	722(62.4)	1624(94.6)	<0.001
-Ex-smoker	250(8.7)	211(18.3)	39(2.3)	
-Smoker	277(9.6)	223(19.3)	54(3.1)	
**Physical activity**				
-High	513(18.5)	290(25.9)	223(13.5)	<0.001
-Moderate	1140(41.1)	402(35.9)	738(44.7)	
-Light	1119(40.4)	429(38.2)	690(41.8)	
**BMI (Kg/m**^**2**^**)**	28.0±4.9*	27.4±4.5	28.5±5.1	<0.001
**Body weight status**				
-Normal weight	792(27.5)	339(29.3)	453(26.3)	<0.001
-Overweight	1210(42.0)	545(47.1)	665(38.6)	
-Obese	878(30.5)	274(23.7)	604(35.1)	
**Waist circumference (cm)**	94.6±12.5*	95.2±11.7	94.2±12.9	0.031
**FBS (mg/dl)**	96.0±25.0	97.6±26.9	94.9±23.7	0.006
**2 hour blood sugar**	117±40.6*	117±43.7	116±38.2	0.712
**Diabetes** (Yes)	376(13.3)	135(11.8)	241(14.3)	0.062
**Total cholesterol (mg/dl)**	186±39.2*	184±38.3	188±39.8	0.023
**HDL (mg/dl)**	47.3±11.2*	42.3±9.5	50.6±11.0	<0.001
**TG (mg/dl)**	119(84.0–173.0)	132(94.8–194.3)	112(78.0–158.0)	<0.001
**SBP (mm Hg)**	115±16.8*	118±15.5	113±17.3	<0.001
**DBP (mm Hg)**	76.7±9.9*	78.8±9.8	75.3±9.7	<0.001
**Hypertension** (Yes)	636(22.2)	255(22.1)	381(22.2)	0.927
**MetS (**Yes**)**	1180 (41.0)	494 (42.7)	686 (39.9)	0.142
**Obesity phenotypes**				
-Normal weight & normal metabolic status	672(23.3)	275(23.7)	397(23.1)	<0.001
-Overweight & normal metabolic status	697(24.2)	279(24.1)	418(24.3)	
-Obese & normal metabolic status	296(10.3)	95(8.2)	201(11.7)	
-Normal weight and dysmetabolic status	120(4.2)	64(5.5)	56(3.3)	
-Overweight and dysmetabolic status	513(17.8)	266(23.0)	247(14.3)	
-Obese and dysmetabolic status	582(20.2)	179(15.5)	403(23.4)	

*Mean±SD; BMI: Body mass index; FBS: Fasting blood sugar; HDL: High-density lipoprotein; TG: Triglycerides; SBP: Systolic blood pressure; DBP; Diastolic blood pressure.

**P values refer to the difference between males and females

**Table 2:** Distribution of socio-demographic factors, smoking status and physical activity among obesity phenotypes in men and women.

	Normal metabolic status			Dysmetabolic status			P
	Normal^a^	Overweight^b^	Obese^c^	Normal^d^	Overweight^e^	Obese^f^	
**Men**							
Age (years)**	44.3±17.2*	45.8±14.9	43.8±13.9	56.2±14.5	52.1±14.6	48.3±13.8	<0.001
**Marital status n(%)**							
-Single	89(32.5)	57(20.4)	12(12.6)	8(12.5)	23(8.7)	21(11.7)	<0.001
-Married	180(65.7)	215(77.1)	80(84.2)	56(87.5)	237(89.4)	157(87.7)	
-Divorced/Widowed	5(1.8)	7(2.5)	3(3.2)	0(0)	5(1.9)	1(0.6)	
**Level of education n(%)**							
-Primary	64(23.4)	60(21.6)	21(22.3)	17(26.6)	70(26.3)	50(28.1)	0.010
-Secondary	92(33.6)	102(36.7)	43(45.7)	24(37.5)	117(44.0)	80(44.9)	
-Higher	118(43.1)	116(41.7)	30(31.9)	23(35.9)	79(29.7)	48(27.0)	
**Job status n(%)**							
-Unemployed/housewife	34(12.5)	24(8.6)	6(6.4)	2(3.1)	13(4.9)	9(5.1)	<0.001
- Unemployed, but had other sources of income	50(18.4)	47(16.8)	7(7.4)	19(29.7)	72(27.1)	32(18.1)	
- Employed	188(69.1)	208(74.6)	81(86.2)	43(67.2)	181(68.0)	136(76.8)	
**Smoking status**							
-Never	172(62.5)	178(63.8)	65(69.1)	38(59.4)	161(60.5)	108(60.7)	0.909
-Ex-smoker	49(17.8)	48(17.2)	17(18.1)	14(21.9)	48(18.0)	35(19.7)	
-Smoker	54(19.6)	53(19.0)	12(12.8)	12(18.8)	57(21.4)	35(19.7)	
**Physical activity**							
-High	84(31.5)	82(30.5)	28(29.5)	10(16.1)	53(20.5)	33(19.4)	<0.001
-Moderate	100(37.5)	102(37.9)	33(34.7)	18(29.0)	99(38.4)	50(29.4)	
-Light	83(31.1)	85(31.6)	34(35.8)	34(54.8)	106(41.1)	87(51.2)	
**Women**							
**Age (years)**	36.9±11.6*	44.2±12.3	47.9±11.9	59.7±15.2	55.5±12.8	55.8±10.9	<0.001
**Marital status n(%)**							
-Single	125(31.5)	39(9.4)	8(4.0)	2(3.6)	10(4.1)	10(2.5)	<0.001
-Married	251(63.2)	347(83.6)	168(83.6)	42(76.4)	184(74.8)	327(81.1)	
-Divorced/Widowed	21(5.3)	29(7.0)	25(12.4)	11(20.0)	52(21.1)	66(16.4)	
**Level of education n(%)**							
-Primary	32(8.1)	79(18.9)	92(45.8)	31(55.4)	120(48.6)	218(54.2)	<0.001
-Secondary	143(36.0)	189(45.3)	70(34.8)	17(30.4)	95(38.5)	139(34.6)	
-Higher	222(55.9)	149(35.7)	39(19.4)	8(14.3)	32(13.0)	45(11.2)	
**Job status n(%)**							
-Unemployed/housewife	251(63.2)	308(73.7)	157(78.1)	38(67.9)	174(70.4)	318(78.9)	<0.001
- Unemployed, but had other sources of income	14(3.5)	27(6.5)	16(8.0)	16(28.6)	49(19.8)	61(15.1)	
- Employed	132(33.2)	83(19.9)	28(13.9)	2(3.6)	24(9.7)	24(6.0)	
**Smoking status**							
-Never	365(91.9)	392(94.0)	190(95.0)	55(98.2)	235(95.9)	387(96.3)	0.145
-Ex-smoker	11(2.8)	9(2.2)	6(3.0)	0(0)	4(1.6)	9(2.2)	
-Smoker	21(5.3)	16(3.8)	4(2.0)	1(1.8)	6(2.4)	6(1.5)	
**Physical activity**							
-High	55(14.3)	72(17.7)	25(13.0)	8(15.1)	32(13.7)	31(8.1)	0.001
-Moderate	176(45.8)	193(47.4)	94(49.0)	22(41.5)	92(39.3)	161(42.3)	
-Light	153(39.8)	142(34.9)	73(38.0)	23(43.4)	110(47.0)	189(49.6)	

*Mean±SD

** Post hoc test for age; in women: a<b&c&d&f; b<d&e&f; c<d&e&f, in men: d>a&b&c&f, d>a&b&c

Mean HRQoL scores were compared among different obesity phenotypes after adjusting for age, marital status, level of education, job status and leisure time physical activity (Table 3). Results showed that in both men and women, mean HRQoL scores were significantly different among obesity phenotypes in physical subscales, including physical functioning, role physical, general health and physical component summary (PCS) scores (p<0.05), although no significant differences were observed in mental subscales or the mental component summary (MCS) scores.

**Table 3:** SF-12 scores in different obesity phenotypes in men and women.

	Normal metabolic status			Dysmetabolic status			P
	Normal	Overweight	Obese	Normal	Overweight	Obese	
**Men**							
-Physical Function	90.9±30.8	91.3±31.7	91.4±24.2	92.9±23.4	87.3±31.7	84.8±28.5	**0.001**
-Role Physical	84.9±32.5	88.0±33.4	85.8±25.5	86.4±24.6	83.9±33.5	81.3±30.1	**0.030**
-Bodily pain	85.9±32.3	88.5±33.3	86.9±25.4	86.3±24.5	84.7±33.3	87.3±29.9	0.392
-General Health	54.5±34.4	55.3±35.4	57.2±26.9	48.2±26.1	51.3±35.4	45.9±31.8	**<0.001**
**PCS**	51.7±10.7	52.6±11.0	52.7±8.4	51.7±8.1	50.8±11.1	49.9±9.9	**<0.001**
-Vitality	64.3±38.5	67.0±39.7	67.3±30.2	64.6±29.2	63.4±39.7	62.9±35.7	0.381
-Social Function	77.5±38.5	77.5±39.6	77.7±30.2	79.9±29.2	75.5±39.7	76.1±35.7	0.774
-Role Emotional	78.7±33.9	77.6±34.9	73.9±26.6	76.9±25.7	75.3±34.9	77.2±31.4	0.376
-Mental Health	67.8±32.0	68.2±32.9	67.8±25.1	67.5±24.3	68.0±32.9	65.9±29.7	0.919
**MCS**	47.0±15.7	46.9±16.1	46.3±12.3	46.8±11.9	46.6±16.1	46.7±14.5	0.988
**Women**							
-Physical Function	85.2±31.5	83.7±31.9	80.5±29.3	82.8±26.8	83.4±29.5	73.3±33.4	**<0.001**
-Role Physical	78.3±28.9	77.6±29.2	75.3±26.9	73.2±24.7	75.6±27.1	71.8±30.6	**0.018**
-Bodily pain	76.4±29.9	78.1±30.2	77.3±27.7	79.3±25.4	76.9±27.9	74.4±31.6	0.401
-General Health	49.9±25.5	50.8±25.8	46.9±23.7	41.1±21.7	45.5±23.9	43.1±26.9	**<0.001**
**PCS**	49.3±10.0	48.9±10.1	48.1±9.3	48.1±8.5	48.2±9.4	45.8±10.6	**<0.001**
-Vitality	61.6±32.5	65.5±32.8	62.5±30.2	56.5±27.7	64.3±30.4	61.5±34.4	0.103
-Social Function	78.3±32.5	80.6±32.8	75.9±30.1	78.2±27.7	76.6±30.4	78.9±34.4	0.352
-Role Emotional	68.9±29.4	71.5±29.7	67.6±27.3	69.6±25.1	70.1±27.5	69.0±31.1	0.518
-Mental Health	65.9±27.1	67.6±27.4	66.3±25.2	60.5±23.1	65.3±25.4	64.9±28.7	0.344
**MCS**	45.7±13.4	47.3±13.5	45.9±12.4	44.4±11.4	46.1±12.5	46.9±14.1	0.218

Data are presented as Mean±SD; SF-12 scores are adjusted for age, marital status, level of education, job status and leisure time physical activity.

PCS: Physical component summary; MCS: Mental component summary.

Table 4 shows the odds of reporting poor physical and mental HRQoL for metabolic status, weight status and different obesity phenotypes in men and women separately. Considering metabolic status, after adjusting for confounding variables, the odds of reporting poor physical HRQoL were significantly higher in both men (OR: 2.135, 95% CI:1.450–3.143; p<0.001) and women (OR: 1.826, 95% CI:1.282–2.600; p = 0.001) with dysmetabolic status, compared to their counterparts with normal metabolic status. For weight status, only obese women were more likely to report poor physical HRQoL, compared to their normal weight counterparts (OR: 2.092, 95% CI: 1.318–3.321; p = 0.002); overweight women were less likely to report poor mental HRQoL, compared to their normal weight counterparts (OR: 0.656, 95% CI: 0.448–0.962; p<0.05). In terms of obesity phenotypes, after adjusting for confounding variables, the odds of reporting poor physical HRQoL was significantly higher in men (OR:1.960, 95% CI:1.037–3.704; p<0.05) and women (OR: 2.887, 95% CI:1.674–4.977; p<0.001) with obese-dysmetabolic status, compared to their counterparts with normal weight-normal metabolic status. In addition, in women the odds of reporting poor mental HRQoL was significantly lower in women with the overweight-normal metabolic status phenotype (OR:0.638, 95%CI: (0.415–0.981); p<0.05) compared to their counterparts with a normal weight-normal metabolic phenotype.

**Table 4:** Odds ratios and 95% confidence intervals for poor HRQoL among men and women.

	Phenotype	PCS	P value	MCS	P value
		OR (95%CI)		OR (95%CI)	
**Men**					
Model 1	- Normal metabolic status	(Ref.)		(Ref.)	
	- Dysmetabolic status	2.753(1.960–3.867)	**<0.001**	0.850(0.612–1.181)	0.332
Model 2	- Normal metabolic status	(Ref.)		(Ref.)	
	- Dysmetabolic status	2.135(1.450–3.143)	**<0.001**	1.108(0.761–1.614)	0.593
Model 1	-Normal weight	(Ref.)		(Ref.)	
	-Overweight	1.151(0.781–1.697)	0.476	0.874(0.593–1.287)	0.494
	-Obese	1.421(0.908–2.222)	0.124	0.995(0.638–1.552)	0.982
Model 2	-Normal weight	(Ref.)		(Ref.)	
	-Overweight	0.999(0.635–1.572)	0.997	1.053(0.682–1.624)	0.816
	-Obese	1.135(0.672–1.918)	0.636	1.113(0.674–1.837)	0.676
Model 1	-Normal weight & normal metabolic status	(Ref.)		(Ref.)	
	-Overweight & normal metabolic status	0.827(0.509–1.344)	0.443	0.892(0.558–1.425)	0.632
	-Obese & normal metabolic status	0.681(0.347–1.336)	0.264	1.014(0.523–1.965)	0.968
	-Normal weight & dysmetabolic status	2.353(1.040–5.322)	**0.040**	0.714(0.312–1.634)	0.425
	-Overweight & dysmetabolic status	2.207(1.353–3.598)	**0.002**	0.761(0.470–1.233)	0.267
	-Obese & dysmetabolic status	2.724(1.574–4.714)	**<0.001**	0.910(0.544–1.523)	0.720
Model 2	-Normal weight & normal metabolic status	(Ref.)		(Ref.)	
	-Overweight & normal metabolic status	0.754(0.430–1.322)	0.324	0.952(0.563–1.608)	0.854
	-Obese & normal metabolic status	0.570(0.267–1.218)	0.147	1.202(0.587–2.458)	0.615
	-Normal weight & dysmetabolic status	1.522(0.610–3.799)	0.368	0.841(0.334–2.117)	0.713
	-Overweight & dysmetabolic status	1.599(0.906–2.824)	0.105	1.154(0.672–1.981)	0.604
	-Obese & dysmetabolic status	1.960(1.037–3.704)	**0.038**	1.116(0.624–1.998)	0.711
**Women**					
Model 1	- Normal metabolic status	(Ref.)		(Ref.)	
	- Dysmetabolic status	4.246(3.179–5.669)	**<0.001**	0.875(0.668–1.147)	0.334
Model 2	- Normal metabolic status	(Ref.)		(Ref.)	
	- Dysmetabolic status	1.826(1.282–2.600)	**0.001**	1.046(0.756–1.446)	0.788
Model 1	-Normal weight	(Ref.)		(Ref.)	
	-Overweight	2.082(1.439–3.011)	**<0.001**	0.590(0.415–0.838)	**0.003**
	-Obese	5.252(3.597–7.670)	**<0.001**	0.725(0.508–1.035)	0.076
Model 2	-Normal weight	(Ref.)		(Ref.)	
	-Overweight	1.187(0.763–1.847)	0.446	0.656(0.448–0.962)	**0.031**
	-Obese	2.092(1.318–3.321)	**0.002**	0.766(0.511–1.149)	0.197
Model 1	-Normal weight & normal metabolic status	(Ref.)		(Ref.)	
	-Overweight & normal metabolic status	1.957(1.259–3.041)	**0.003**	0.588(0.393–0.879)	**0.010**
	-Obese & normal metabolic status	3.429(2.040–5.764)	**<0.001**	0.954(0.589–1.546)	0.850
	-Normal weight & dysmetabolic status	6.858(2.772–16.966)	**<0.001**	1.402(0.566–3.473)	0.466
	-Overweight & dysmetabolic status	4.742(2.887–7.788)	**<0.001**	0.653(0.416–1.025)	0.064
	-Obese & dysmetabolic status	9.917(6.292–15.629)	**<0.001**	0.666(0.446–1.025)	**0.047**
Model 2	-Normal weight & normal metabolic status	(Ref.)		(Ref.)	
	-Overweight & normal metabolic status	1.185(0.720–1.949)	0.505	0.638(0.415–0.981)	**0.041**
	-Obese & normal metabolic status	1.721(0.951–3.113)	0.073	0.984(0.582–1.664)	0.952
	-Normal weight & dysmetabolic status	1.838(0.629–5.371)	0.266	1.795(0.654–4.930)	0.256
	-Overweight & dysmetabolic status	1.650(0.911–2.988)	0.099	0.866(0.512–1.462)	0.589
	-Obese & dysmetabolic status	2.887(1.674–4.977)	**<0.001**	0.782(0.485–1.261)	0.313

Model 1: Unadjusted. Model 2: Adjusted for age, marital status (ref = married), level of education (ref = higher), job status (ref = employed) and physical activity (ref = high). PCS: Physical component summary; MCS: Mental component summary.

## Discussion

This study showed that among different obesity phenotypes, only the obese-dysmetabolic phenotype is associated with poor physical HRQoL in both men and women. However, except for overweight-normal metabolic women, who were less likely to report poor mental HRQoL, none of the phenotypes were associated with mental HRQoL in either gender. Furthermore, a comparison of total and subscale scores of HRQoL indicated that in both men and women, except for bodily pain, other physical subscales as well as PCS scores differed significantly among obesity phenotype groups.

Our findings regarding significant associations between obesity phenotypes and physical HRQoL, are consistent with results of previous studies conducted among other populations, confirming that HRQoL among different obesity phenotypes is negatively affected only in the physical but not the mental domains; in this regard a prospective cohort study in Spain revealed that obese individuals with metabolic abnormalities are more likely to report poor physical HRQoL, compared to their normal weight-normal metabolic counterparts. However, the same results have been observed for healthy obese individuals in the mentioned population, emphasizing the role of weight rather than metabolic status in Spanish adults [38]. In an obese Scottish population, results revealed that overall utility score was diminished in individuals regardless of their metabolic comorbidities [37]. In addition, another study from Korea indicated that, while mobility and physical functioning are severely affected in obese women, regardless of their metabolic status, only metabolically abnormal and normal weight men are more likely to report poor HRQoL in these physical domains [39].

The aforementioned studies considered weight status to be a more significant factor associated with impaired HRQoL, especially in women [37–39], results consistent with our findings highlighting the importance of obesity in predicting poor self-assessment of health in Iranian women. However, in men, regardless of weight status, metabolic status plays a significant role to predict physical wellbeing, a result in accordance with our previous findings, which revealed higher prognostic values for metabolic abnormalities in predicting CVD events in comparison with weight status per se in the TLGS population [47]. In the current study, the significant effect of co-incidence of the obesity and dysmetabolic status on poor physical HRQoL was observed in both genders; it seems that, in comparison to the separate effects of weight and metabolic status on PCS, the co-incidence would have stronger effect in women.

Based on the current results, except for overweight-normal metabolic women, who reported better mental HRQoL than their normal weight-normal metabolic counterparts, none of the obesity phenotypes studied showed significant associations with mental domains of HRQoL in either gender, a finding in agreement with several previous studies suggesting that overweight individuals compared to their normal weight counterparts, perceived better mental HRQoL [22,24,28,48]. Although there is no clear-cut explanation for the positive associations observed between overweight and mental HRQoL in the current and other previous studies; there are data in women on the effect of ideal body weight on their physical, psychological, and social wellbeing that may be influenced by their socio-demographic status [49], economic conditions [48,50], preferences of significant others [51,52] and other cultural factors and media exposures [53,54]. In this regard, a recent international study conducted in 26 countries demonstrated that compared to countries with high socio-economic status (SES), heavier body weight was preferred more in lower socio-economic societies [50]; body fat in these populations is sometimes considered as a symbol of security and wealth among these populations, which could possibly explain such findings [48,50]. It has also been suggested that in populations with low SES, women who were older, heavier and less exposed to Western media are preferred and are hence more satisfied with their heavier appearance [50]. On the other hand, other data available emphasize weight misperception as another contributor to this high mental HRQoL in overweight women [55,56]; these women hence overestimate their height and underestimate their weight which results in inaccurate estimation of data on their excessive weight [57,58], as a result of which overweight women consider themselves as normal weight and are probably satisfied with their weight status, and hence not interested in joining weight-loss programs [59].

This study has both strengths and limitations. Among the former, to the best of our knowledge, this is one of the first efforts to investigate the association between HRQoL as a subjective patient-centered outcome among different obesity phenotypes in a large Middle-Eastern adult population. Current data reveals the probability of CVD events in adult TLGS participants with different obesity phenotypes, and our subjective results provide a comprehensive understanding of the effects of obesity and its related cardio-metabolic risk factors on the individual’s health. However, this study does have some limitations; first, the cross-sectional design of this study limits our ability to draw conclusions regarding the causal effect of obesity phenotypes on HRQoL; second, our results are limited to a specific urban population in Tehran and cannot be generalized to all Tehranian adults or to those adults residing in rural sites and other cities; last but not least, there were many confounders that could affect HRQoL, and the observed associations between obesity phenotypes and HRQoL might be a result of unmeasured variables.

In conclusion, while metabolic status was associated with poor physical HRQoL in both genders, weight status only influenced physical wellbeing in women. However, among different obesity phenotypes, only obese dysmetabolic individuals were more likely to report poor physical HRQoL, findings indicating a similar pattern in both genders, indicating that despite the high risk for developing cardiovascular outcomes, normal/overweight individuals with dysmetabolic status may not feel the urgent need for preventive measures. These findings could be valuable in identifying vulnerable groups and prioritizing strategies in related health promotion programs.

## Supporting information

S1 Table The minimal data set. (SAV)
